# Type V collagen-induced nasal tolerance prevents lung damage in an experimental model: new evidence of autoimmunity to collagen V in COPD

**DOI:** 10.3389/fimmu.2024.1444622

**Published:** 2024-09-05

**Authors:** Fabíola Santos Zambon Robertoni, Ana Paula Pereira Velosa, Luana de Mendonça Oliveira, Francine Maria de Almeida, Lizandre Keren Ramos da Silveira, Zelita Aparecida de Jesus Queiroz, Thays de Matos Lobo, Vitória Elias Contini, Camila Machado Baldavira, Solange Carrasco, Sandra de Morais Fernezlian, Maria Notomi Sato, Vera Luiza Capelozzi, Fernanda Degobbi Tenorio Quirino dos Santos Lopes, Walcy Paganelli Rosolia Teodoro

**Affiliations:** ^1^ Division of Rheumatology, Faculdade de Medicina da Universidade de São Paulo, São Paulo, Brazil; ^2^ Laboratory of Dermatology and Immunodeficiencies, Laboratório de Investigação Médica (LIM)-56, Department of Dermatology, Tropical Medicine Institute of São Paulo, University of São Paulo Medical School, São Paulo, Brazil; ^3^ Department of Clinical Medicine, Laboratory of Experimental Therapeutics, Laboratório de Investigação Médica (LIM)-20, Faculdade de Medicina da Universidade de São Paulo, São Paulo, Brazil; ^4^ Department of Pathology, Faculdade de Medicina da Universidade de São Paulo, São Paulo, Brazil

**Keywords:** chronic obstructive pulmonary disease, cigarette smoking, pulmonary emphysema, animal models, autoimmunity, collagen type V, regulatory T cell, immune tolerance

## Abstract

**Background:**

Chronic obstructive pulmonary disease (COPD) has been linked to immune responses to lung-associated self-antigens. Exposure to cigarette smoke (CS), the main cause of COPD, causes chronic lung inflammation, resulting in pulmonary matrix (ECM) damage. This tissue breakdown exposes collagen V (Col V), an antigen typically hidden from the immune system, which could trigger an autoimmune response. Col V autoimmunity has been linked to several lung diseases, and the induction of immune tolerance can mitigate some of these diseases. Evidence suggests that autoimmunity to Col V might also occur in COPD; thus, immunotolerance to Col V could be a novel therapeutic approach.

**Objective:**

The role of autoimmunity against collagen V in COPD development was investigated by analyzing the effects of Col V-induced tolerance on the inflammatory response and lung remodeling in a murine model of CS-induced COPD.

**Methods:**

Male C57BL/6 mice were divided into three groups: one exposed to CS for four weeks, one previously tolerated for Col V and exposed to CS for four weeks, and one kept in clean air for the same period. Then, we proceeded with lung functional and structural evaluation, assessing inflammatory cells in bronchoalveolar lavage fluid (BALF) and inflammatory markers in the lung parenchyma, inflammatory cytokines in lung and spleen homogenates, and T-cell phenotyping in the spleen.

**Results:**

CS exposure altered the structure of elastic and collagen fibers and increased the pro-inflammatory immune response, indicating the presence of COPD. Col V tolerance inhibited the onset of emphysema and prevented structural changes in lung ECM fibers by promoting an immunosuppressive microenvironment in the lung and inducing Treg cell differentiation.

**Conclusion:**

Induction of nasal tolerance to Col V can prevent inflammatory responses and lung remodeling in experimental COPD, suggesting that autoimmunity to Col V plays a role in COPD development.

## Highlights

- Autoimmunity to type V collagen is involved in COPD physiopathology.
- Immunotolerance to type V collagen can prevent emphysema development.

## 1 Introduction

Chronic obstructive pulmonary disease (COPD) is a highly prevalent disease and one of the most important public health problems worldwide (1). It is characterized by extracellular matrix remodeling in the airways and alveolar parenchyma due to a chronic inflammatory process (2) and is mainly caused by exposure to cigarette smoke (CS) (3). COPD patients suffer from dyspnea and fatigue, which are due to impaired respiratory mechanics, gas exchange abnormalities, and deconditioning-related dysfunction of the peripheral muscles that affect their capacity to accomplish daily activities as well as their overall quality of life and life expectancy. (3). The frequent progressive worsening of the disease (4) eventually leads to exacerbations and hospitalizations that are associated with a poor prognosis (5). Pharmacotherapy can alleviate COPD symptoms, reduce exacerbations, and enhance overall health and exercise tolerance (3). Existing therapies, aside from smoke cessation, cannot consistently prevent disease progression and can cause many adverse health effects (6), such as cardiac rhythm disturbance, dryness of mouth, headaches, insomnia and nausea caused by bronchodilators, oral candidiasis, hoarse voice, skin bruising and pneumonia caused by inhaled corticosteroids (3), Therefore, better understanding of the mechanisms that underlie the development and progression of COPD is essential to help improve the management of this highly challenging and complex disease.

COPD is a multifactorial disease, and its pathophysiology has been linked to a number of mechanistic ideas (7), with increasing evidence that autoimmunity plays an essential role in its pathogenesis (8). Breakdown products from the extracellular matrix (such as collagen and elastin fragments) can activate adaptive immune responses in COPD patients’ lungs, including cytotoxic CD8^+^ T cells, T helper (Th)1 and Th17 CD4^+^ T cells, and B-cell responses leading to autoantibodies (7). Recent research has demonstrated other autoimmunity components associated with COPD. The serum antibody titer against carbonyl-modified proteins, resulting from the oxidation of various amino acid residues, is significantly elevated in COPD patients (GOLD 3) compared with healthy controls, which could lead to an autoimmune response (9). B cell-activating factor of tumor necrosis factor family (BAFF) overexpression is associated with autoimmune diseases, and it was found to be up-regulated in B cells in pulmonary lymphoid follicles (LFs), in blood and bronchoalveolar lavage samples from COPD patients (10). Blood samples collected from 2,396 individuals in the COPDGene study, indicated that interferon (IFN)-stimulated genetic signatures from bronchial brushings and peripheral blood were associated with exacerbations, lung function, and airway wall thickness. Since these samples were obtained during periods of stable disease, this genetic signature identified is likely the result of sustained autoimmune responses (11).

Damage caused directly by cigarette smoke and inflammation results in the degradation of collagen and the release of its fragments into the systemic circulation, (12) which could trigger autoimmune responses. In this sense, researchers have conducted studies to evaluate serological markers as potential diagnostic and prognostic markers of COPD (13–17). It has been proposed that fragments of types I, III, and V are the most effective in distinguishing between mild COPD patients and healthy controls. (12) A previous study showed that immunity against type V collagen (Col V) may play a role in COPD, as markers related to this process have been found in the serum of patients, especially during disease exacerbations (13). Furthermore, the presence of anti-collagen V antibody in an ELISA assay, and collagen V-specific Th1 immune responses (IFNγ) (EliSpot assay performed on isolated peripheral blood mononuclear cells) has been demonstrated in the peripheral blood of patients with COPD and smokers, suggesting that Col V-specific autoimmunity is associated with cigarette smoking history and may be involved in the pathogenesis of COPD (17).

Col V is a fibrillar collagen that is a minor component of the ECM and is found in the lung interstitium and capillary basement membranes (18). Usually, it is embedded within heterotypic collagen I/III fibrils (19–21). Due to its location and immunogenic and antigenic properties, Col V is considered a sequestered antigen that remains hidden from the immune system under normal conditions (22). However, in cases of tissue damage, such as CS-induced ECM lung injury, Col V epitopes may be exposed to the immune system, which can turn them into potential self-antigens (23, 24).

The role of autoimmunity against Col V has been explored in various diseases in recent decades, both in murine models and humans. Col V-specific autoimmunity is involved in several pathological processes, including some lung diseases, such as idiopathic pulmonary fibrosis (IPF) and asthma (23, 25–29). Conversely, different approaches to inducing immunological tolerance to Col V have proven effective in decreasing lung allograft rejection (23, 24, 30–32), reducing atherosclerotic plaque (33), preventing induced airway hyperresponsiveness (22), and mitigating pulmonary fibrosis (27, 34).

Taken together, this evidence suggests that in COPD, autoimmunity against Col V may play a role in the pathophysiology of the disease, and the induction of tolerance to this collagen may protect against ECM lung damage. Thus, our objective was to investigate this hypothesis by assessing whether Col V-induced tolerance interferes with the course of emphysema development in a short-term murine model of CS-induced COPD.

## 2 Methods

### 2.1 Experimental groups

Male C57BL/6 mice (aged 6–8 weeks and weighing 20–25 g) were divided at random into three groups: first, exposed to CS for four weeks (CS group); second, previously tolerated for Col V and exposed to CS for the same four weeks (CS/Tol group); and third, maintained under room air conditions for the same time intervals as the controls (CT group). All the animals received proper care according to the National Research Council’s Guide for the Care and Use of Laboratory Animals (2011). Our protocol was approved by the Ethics Committee on the Use of Animals from the Faculty of Medicine of the University of São Paulo under protocol number 1200/2018 (São Paulo, Brazil). To properly process the biological samples, the animals were divided into two groups: first, functional and morphological analyses of the lungs were performed; second, cell and cytokine profile analyses were performed.

### 2.2 Type V collagen nasal tolerance protocol

Induction of tolerance to Col V was achieved by daily nasal administration of 20 µl of Col V solution (0.5 mg/ml; Human Placenta Collagen, Bornstein and Traub Type V powder, Sigma-Aldrich, USA) to each animal (33), which was repeated for five days during the week before beginning the exposure to cigarette smoke protocol. Boosters at the same dosage were given three times a week for the entire duration of the cigarette smoke exposure protocol. Nasal administration of Col V was performed at least 30 minutes before the first CS exposure of the day, to ensure permeability of the nasal cavity of mice to CS.

### 2.3 CS exposure protocol

The animals were exposed to CS as previously described, in a in a full body inhalation chamber (28 L) (35), with slight modifications according to a more recent evaluation (36). Using a whole-body exposure chamber, animals were exposed to the CS of twenty commercially filtered cigarettes per day (Souza Cruz – BAT, Brazil; 10 mg of tar, 0.8 mg of nicotine, and 10 mg of CO per cigarette) (35). A 60-minute full body exposure to CS from 10 cigarettes was performed twice a day for five days/week for four weeks. The flow rate was set such that the carbon monoxide (CO) levels inside the chamber were maintained at approximately 350 parts per million (ppm). The control groups were maintained under clean room air conditions.

### 2.4 Respiratory mechanics evaluation

On the day following the last exposure to cigarette smoke, the first group of mice was deeply anesthetized by an intraperitoneal injection of 50 mg/kg sodium thiopental, tracheostomized and mechanically ventilated (n=7–10 per group) using a flexiVent small animal ventilator (SCIREQ, Montreal, QC, Canada). Pulmonary function assessment was performed using the forced oscillatory technique and a constant phase model (37), and values for the parameters of airway resistance (Raw), tissue damping (Gtis), and tissue elastance (Htis) were obtained.

### 2.5 Total cell and macrophage measurements in BALF

Immediately after lung mechanics assessment, the mice were euthanized by abdominal aortic exsanguination, and bronchoalveolar lavage fluid (BALF) samples were collected. The animals’ lungs were washed two times with 1 ml of phosphate-saline buffer (PBS) and infused into the lungs through the tracheal cannula, after which the fluid was recovered for subsequent analyses. The fluid was centrifuged at 1000 rpm for 8 min at 5°C, and the cell pellet was resuspended in 300 μl of physiological saline for total cell counting using a Neubauer hemocytometer chamber. Aliquots of the cell suspension were centrifuged on glass slides using a cytocentrifuge and stained with Diff-Quik (Medion Diagnostics, Dündingen, Switzerland). Differential cells (300 cells/slide) were evaluated by light microscopy with an immersion objective (38).

### 2.6 Lung preparation for histological analyses

After BALF recovery, the lungs were removed and fixed at constant pressure (20 cmH_2_O) using 10% buffered formalin infused through the trachea for 24 h (35). Then, the lungs were paraffin-embedded and cut into 5-μm-thick sections.

#### 2.6.1 Morphometry

Lung tissue sections were stained with hematoxylin and eosin (H&E) to evaluate the mean linear intercept (Lm), which is used as an indicator of the mean diameter of airspaces, as described previously (39). The Lm was analyzed in a blinded fashion, and 20 randomly selected nonoverlapping fields of lung parenchyma were assessed for each slide at 200x magnification via standard light microscopy using an eyepiece with a known area attached to the ocular lens of the microscope (40). The same lung slides were scanned using a high-resolution digital slide scanner (Pannoramic 250 SCAN, 3DHISTECH Ltd., Budapest, HU). Subsequently, using SlideViewer 2.5 software (3DHISTECH Ltd., Budapest, Hungary), 5 to 10 random fields in the region of the peribronchovascular axis were acquired at a 20x zoom level. To quantify edema, the stereological method of counting points was applied (41) using image analysis software (Image-Pro Plus 6.0 for Windows, Media Cybernetics, Inc., Silver Spring, MS, USA). The number of positive hits for edema was counted and divided by the respective peribronchovascular total area calculated by the software (the results are expressed as +/μm^2^).

#### 2.6.2 Histochemistry

For the identification of total elastic fibers on the lung tissue, sections were stained using the modified Weigert resorcin-fuchsin method, with prior oxidation by Oxone, as previously described (42). This method stains in purple all components of the elastic system, including fully developed elastic, oxytalan, and eulanin fibers, not allowing the identification of the components separately.

#### 2.6.3 Immunohistochemistry

Lung tissue sections were immunostained using the biotin-streptavidin peroxidase method. The following primary antibodies were used: rat monoclonal anti-MAC-2 (CL8942AP, Cedarlane Labs, 1:25000), rabbit polyclonal anti-TGFβ1 (sc-146, Santa Cruz Biotechnology, 1:1500), rabbit polyclonal anti-FOXP3 (sc-28705, Santa Cruz Biotechnology, 1:100), rabbit polyclonal anti-IL-10 (BS20373R, Bioss Antibodies Inc., 1:3000) and rabbit polyclonal anti-IL-17 (sc-7927, Santa Cruz Biotechnology, 1:100). To complete the reactions, specific secondary antibodies (VECTASTAIN^®^ Elite^®^ ABC Kit - Vector Laboratories, Burlingame, CA, USA) were used. Then, the sections were stained with 3,3’-diaminobenzidine (DAB; Sigma-Aldrich Chemie, Steinheim, Germany) and counterstained with Harris’s hematoxylin (Merck, Darmstadt, Germany). Some slices were not incubated with primary antibodies and served as negative controls.

#### 2.6.4 Immunofluorescence

The lung slices were incubated with a rabbit anti-collagen type I antibody (600-401-103-0.1, Rockland, Limerick, PA, USA, 1:2500) overnight at 4°C and then with a secondary antibody (Alexa 488-conjugated goat anti-rabbit IgG, Invitrogen, Life Technologies, Eugene, OR, USA, 1:200) at room temperature. As a control, PBS was used in place of the primary antibody.

#### 2.6.5 Immunofluorescence for double staining

The lung slices were incubated with rabbit polyclonal anti-FOXP3 (sc-28705, 1:50, Santa Cruz Biotechnology, CA, USA) and mouse monoclonal anti-IL-10 (sc-8438, 1:50, Santa Cruz Biotechnology, CA, USA) and then with Alexa 488-conjugated goat anti-rabbit IgG (1:200, Invitrogen, Eugene, OR, USA) and Alexa 546-conjugated donkey anti-mouse IgG (1:200, Invitrogen, Eugene, OR, USA) at room temperature. The nuclei were then counterstained with DAPI (Molecular Probes, Invitrogen, Eugene, OR, USA).

#### 2.6.6 Image analysis

Images of all stained slices were acquired with a photographic camera (QColor 5, Olympus Co., St. Laurent, Quebec, Canada) coupled to a microscope (Olympus BX-51, Olympus Corporation, Tokyo, Japan) and sent to a computer through a scanning system (Oculus TCX, Coreco, Inc., St. Laurent, Quebec, Canada). For all the stains performed, ten random and nonoverlapping fields were evaluated for each animal by a researcher blinded to the study groups. To quantify the elastic fibers (400x magnification) and immunostained cells (1000x magnification), the stereological method of counting points was used (41). The results for elastic fibers are given as the proportion of points with positive staining to the total number of points touching the parenchyma, and the results for immunostaining are given as the proportion of points with positive staining to the total number of points touching the parenchyma. The number of type I collagen fibers (400x magnification) was determined as the ratio of the immunostained area to the total parenchyma area. Measurements were made using Image-Pro Plus 6.0 software (Media Cybernetics, Inc., Silver Spring, MS, USA). The results of each slide are presented as the average of the percentages of all evaluated fields.

### 2.7 Immunophenotyping of T cells from mouse spleen by flow cytometry

From the second group of mice, the spleens were harvested for T-cell immunophenotyping. Mice were first anesthetized and euthanized, and the spleen was removed using previously washed and autoclaved dissection tools. Each spleen was dissociated into a single-cell suspension by maceration on a 40 µm nylon cell strainer (BD, San Diego, CA, USA) and placed in Petri dishes containing RPMI 1640 medium (Sigma, USA) supplemented with 10% fetal bovine serum (FBS). The samples were transferred to tubes, centrifuged, washed twice with RPMI 1640 and resuspended in 1 ml of PBS. After splenic cell harvesting, the samples were incubated with Fc blocking solution and stained with a surface antibody mixture containing anti-CD3 (PercP-Cy5; BD Pharmingen, clone 17A2), anti-CD4 (FITC; BD Pharmingen, clone H129.19), anti-CD8 (APC-Cy7; BD Pharmingen, clone 53-6.7), anti-CD25 (APC; BD Pharmingen, clone PC61), and anti-CD44 (BV 605; BD Horizon, clone IM7). Then, the cells were fixed/permeabilized and incubated with anti-FOXP3 V450 (BD Horizon, clone MF23) for intracellular staining. After 30 min, the cells were washed twice with staining buffer, resuspended, and analyzed by flow cytometry. A total of 500,000 events per sample were acquired on an LSRFortessa cytometer (BD Biosciences, USA), and the data were analyzed with FlowJo software (BD, OR, USA).

### 2.8 Preparation of lung and spleen homogenates for cytokine dosage

Spleens were harvested from the first group of animals and lungs from the second group after anesthesia and euthanasia as described above and stored in a -80°C freezer for later preparation of homogenates. After removal, an average of 44 mg of left lung tissue was homogenized in 750 μl of PBS supplemented with a protease inhibitor (SIGMA P2714-1BTL, 1:10 solution), and an average of 33 mg of spleen tissue was homogenized in 500 μl of the same solution with a tissue homogenizer (PowerLyzer^®^ 24, QIAGEN). The homogenates were kept at 4°C and centrifuged at 4000 RCF for 10 min. The supernatants were collected and stored at −80°C before cytokine levels were assessed.

#### 2.8.1 Cytokine dosage by flow cytometry

The levels of IFN-γ, IL-10, IL-17A, IL-6, and TNF cytokines in lung and spleen homogenates were determined by flow cytometry using a commercial kit specific for mice (BD Cytometric Bead Array (CBA) Mouse Enhanced Sensitivity Master Buffer Kit, BD Biosciences, San Diego, CA, USA), and procedures were followed according to the manufacturer’s instructions. The analysis was conducted in a flow cytometer (LSR Fortessa, BD), and the levels were determined using CBA analysis software.

#### 2.8.2 Protein dosage

As fragments of different sizes were used for tissue homogenates, the total protein dosage of each sample was used to correct the cytokine levels found, increasing the accuracy of the analysis. The total protein concentration in the lung and spleen homogenates was measured by using a commercial kit based on bicinchoninic acid (Bicinchoninic Acid Protein Assay Kit, BCA1, Sigma-Aldrich, USA) following the manufacturer’s instructions on a spectrophotometer (Evolution 60S UV-Visible spectrophotometer, Thermo Scientific, Madison, WI, USA).

### 2.9 Statistical analysis

Statistical analyses were performed using GraphPad Prism 8 (GraphPad Software, Inc.). The normality of the data distribution was verified with the Shapiro-Wilk test. To compare the three experimental groups and detect significant differences, one-way ANOVA was performed, followed by the Holm-Sidak multiple comparisons test or Kruskal-Wallis test, followed by Dunn’s multiple comparisons test, depending on the normality of the variables. A p-value of <0.05 was considered significant.

## 3 Results

### 3.1 Immune tolerance to Col V protects the lungs from cigarette smoke-induced harm

#### 3.1.1 Lung morphometry and respiratory mechanics

**Figure 1:**
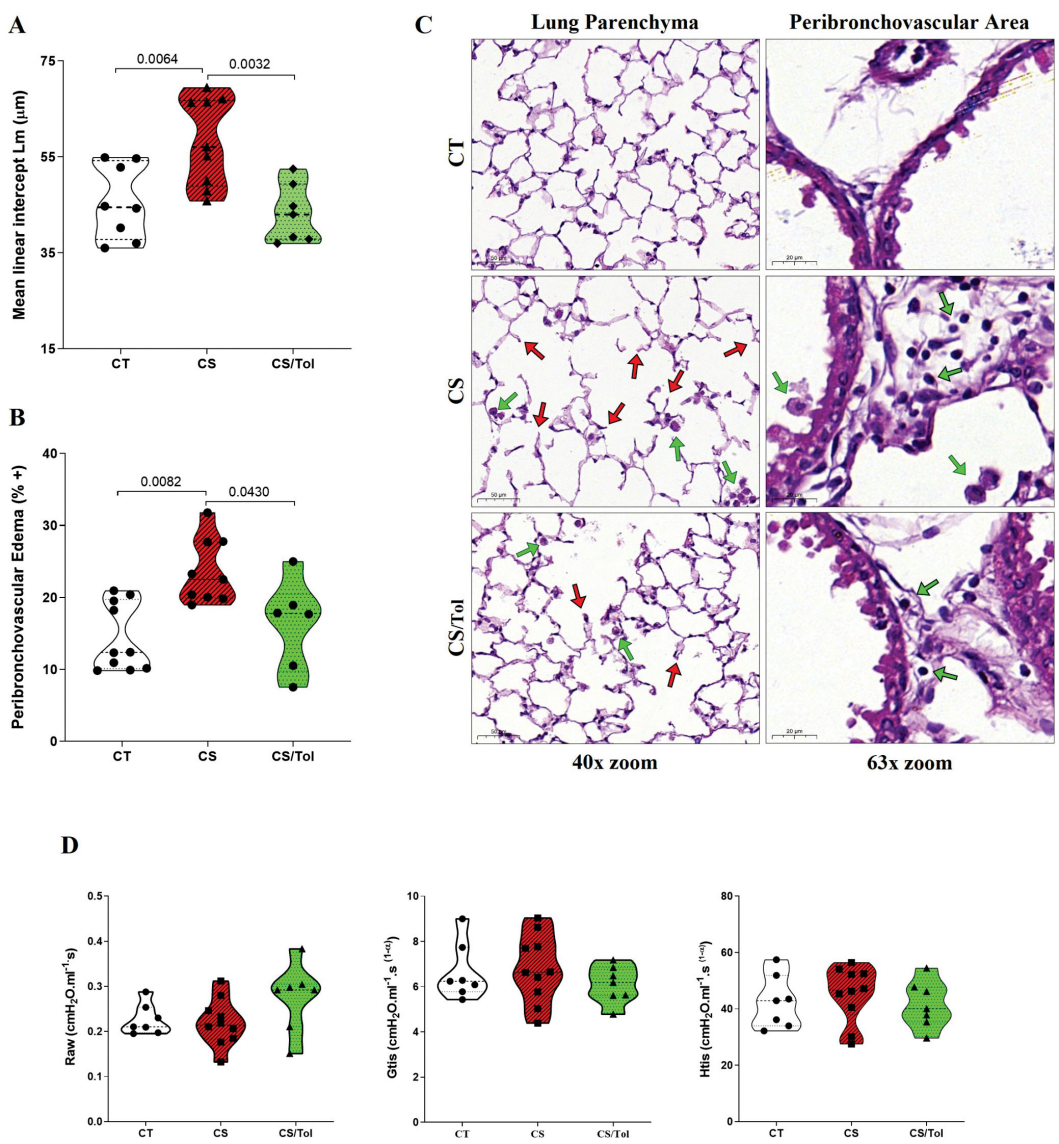
Lung Morphometry and Respiratory Mechanics **(A)** Increased Lm in the Cigarette Smoke (CS) group compared to that in the Control (CT) and Cigarette Smoke/Tolerance (CS/Tol) groups (p=0.0064 and p=0.0032, respectively; one-way ANOVA and Holm-Sidak multiple comparisons test; CT n=8, CS n=9, CS/Tol n=7). **(B)** Increased peribronchovascular edema in the CS group compared to the CT and CS/Tol groups (p=0.0082 and p=0.043, respectively, Kruskal-Wallis and Dunn´s multiple comparisons test; CT n=10, CS n=9, CS/Tol n=6). Violin plot graphs show the frequency distribution of the analyzed data, the median, and the interquartiles. **(C)** Photomicrographs of the lung parenchyma in the CT, CS, and CS/Tol groups (40x magnification) and the peribronchovascular area (63x magnification). H&E staining. Red arrows indicate tissue damage with alveolar enlargement, and green arrows indicate inflammatory cells. **(D)** There was no difference in airway resistance (Raw), tissue damping (Gtis), or tissue elastance (Htis) among the experimental, CT, CS, and CS/Tol groups (one-way ANOVA and Holm-Sidak multiple comparisons test; CT n=7, CS n=10, CS/Tol n=7). Violin plot graphs show the frequency distribution of the analyzed data, the median, and the interquartiles.

Lm was significantly greater in the Cigarette Smoke (CS) group than in the Control (CT) group (**Figure 1A**, p=0.0064), suggesting enlargement of the air spaces and loss of alveolar units. Compared with those in the CS group, the Lm in the Cigarette Smoke/Tolerance (CS/Tol) group significantly decreased (**Figure 1A**, p=0.0032), with values comparable to those in the CT group. Analysis revealed significantly greater edema in the peribronchovascular region in the CS group than in the CT group (**Figure 1B**; p=0.0082), and the level of edema was significantly lower in the CS/Tol group than in the CS group. (**Figure 1B**; p=0.043). Both groups exposed to cigarette smoke had some degree of subacute changes in the lung parenchyma and bronchovascular area, including inflammatory cell infiltration. However, these changes were diffuse and severe only in the CS group (**Figure 1C**). Despite the identified structural changes, respiratory system mechanics evaluation revealed no differences between the experimental groups for any of the evaluated parameters (airway resistance - Raw, tissue damping - Gtis, and tissue elastance - Htis; **Figure 1D**).

#### 3.1.2 Lung matrix composition

**Figure 2:**
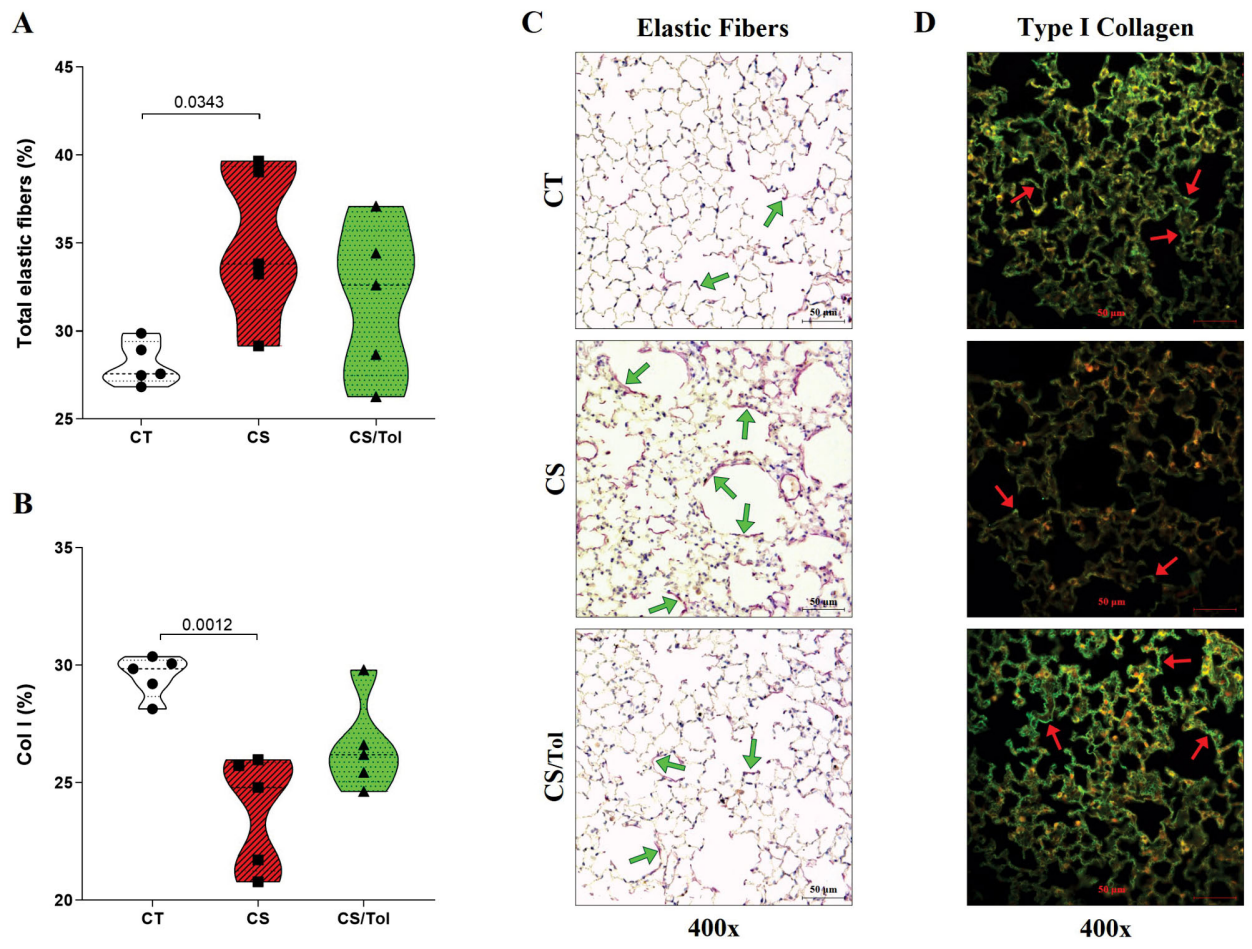
Identification of type I collagen and elastic fibers in the lung parenchyma. **(A)** The proportion of elastic fibers in the CS group was significantly greater than in the CT group (p=0.0393, one-way ANOVA and Holm-Sidak multiple comparisons test; n=6 in each experimental group). **(B)** The proportion of collagen type I fibers was significantly lower in the CS group than in the CT group (p=0.0012, one-way ANOVA and Holm-Sidak multiple comparisons test; n=5 in each experimental group). Violin plot graphs showing the frequency distribution of the analyzed data, the median and the interquartiles. **(C)** Photomicrographs of alveolar parenchyma with modified resorcin-fuchsin immunostaining (400x magnification) in the CT, CS, and CS/Tol groups, showing an increase in elastic fiber breakdown in the CS group reflected by increased staining in purple (green arrows show examples of positive staining). **(D)** Photomicrographs of alveolar parenchyma with type I collagen immunofluorescence staining (400x magnification) in the CT, CS, and CS/Tol groups, show a decrease in those fibers in the CS group (red arrows show examples of positive staining in green).

Histomorphometric evaluation revealed a significant increase in elastic fiber staining in the lung parenchyma only in the CS group compared with the CT group (**Figure 2A**; p=0.0343). There was a significant reduction in the proportion of type I collagen fibers in the CS group compared to that in the CT group (**Figure 2B**; p=0.0012), and there was a tendency toward an increase in the proportion of type I collagen fibers in the CS/Tol group compared to that in the CS group (p= 0.0513). Although the elastic fibers suffered damage in both groups exposed to cigarette smoke, the significant increase in their staining intensity in the CS group reflects greater elastic fiber breakdown in the lung parenchyma in this group, as represented in **Figure 2C**. In **Figure 2D**, the photomicrographs show a decrease in type I collagen immunofluorescence staining in the CS group compared to the CT group.

### 3.2 Regulatory cell profile induced by Col V tolerance

#### 3.2.1 Macrophages in BALF and lung parenchyma

**Figure 3:**
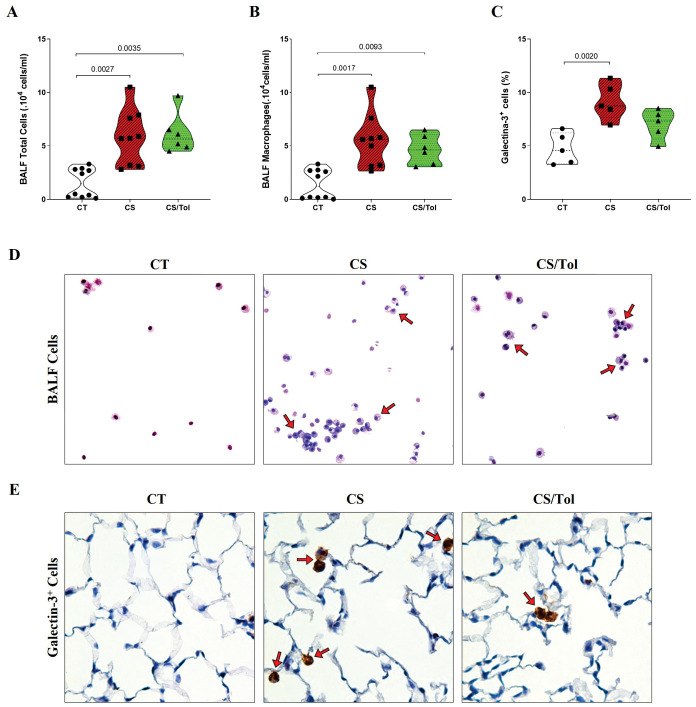
Total cell and macrophage counts in BALF, the proportion of Galectin-3^+^ cells in the lung parenchyma, and representative photomicrographs of total cells in BALF and immunostaining for Galectin-3+ cells. **(A)** There was a significant increase in the number of total BALF cells in the CS and CS/Tol groups compared to that in the CT group (p=0.0027 and p=0.0035, respectively; Kruskal-Wallis test and Dunn’s multiple comparisons test; CT, n=10; CS, n=9; CS/Tol, n=6). **(B)** Differential cell counts revealed a predominance of macrophages among the BALF cells, with significantly greater numbers in the CS and CS/Tol groups than in the CT group (p=0.0017 and p=0.0093, respectively; Kruskal-Wallis and Dunn’s multiple comparisons test; CT, n=10; CS, n=9; CS/Tol, n=6). **(C)** The number of Galectin-3^+^ cells was significantly greater in the CS group than in the C group (p=0.002, one-way ANOVA and Holm-Sidak’s multiple comparisons tests; n=5 in each experimental group). The violin plots show the frequency distributions of the analyzed data. **(D)** Photomicrographs of BALF cells in the CT, CS, and CS/Tol groups (200x magnification; Diff-Quik staining; red arrows show the predominance of macrophages in BALF). **(E)** Photomicrographs of galectin-3 immunostaining in the lung parenchyma in the CT, CS and CS/Tol groups (1000x magnification; red arrows show positive cells in brown).

There was a significant increase in the total number of inflammatory cells in the BALF of the groups exposed to cigarette smoke, CS or CS/Tol compared to that in the BALF of the CT group (**Figure 3A**; p=0.0027 and p=0.0035, respectively). Differential evaluation revealed a predominance of macrophages, whose total number was significantly greater in the CS and CS/Tol groups than in the CT group (**Figure 3B**; p=0.0017 and p=0.0093, respectively). After four weeks of exposure to cigarette smoke, we observed an increase in Galectin-3^+^ cells distributed throughout the lung parenchyma in animals from the CS and CS/Tol groups, but statistical analysis revealed a significant increase in this marker only in the CS group compared to that in the CT group (**Figure 3C**; p=0.0020) and a tendency toward a decrease in this marker in the CS/Tol group in comparison to that in the CS group (p= 0.0721). In **Figures 3D**, **E**, the photomicrographs illustrate the increase in cells in the BALF and the galectin-3 immunostaining in the lung parenchyma, respectively.

#### 3.2.2 IL-17^+^, IL-10^+^, TGFβ^+^ and FOXP3^+^ cells in the lung parenchyma

**Figure 4:**
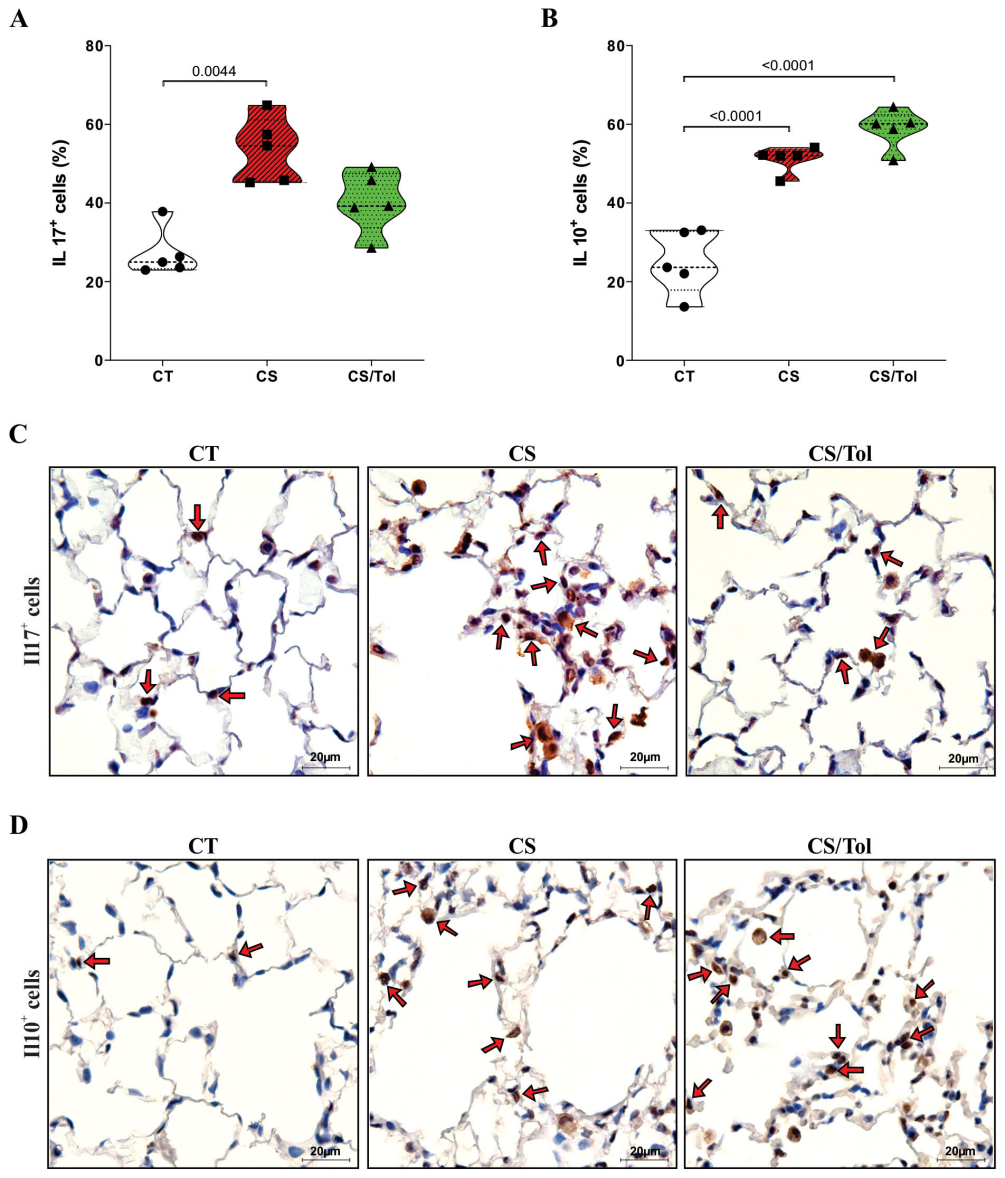
Proportion of IL-17^+^ and IL-10^+^ cells in the lung parenchyma and representative photomicrographs of immunostaining for IL-17^+^ and IL-10^+^ cells. **(A)** There was a significant increase in the number of IL-17^+^ cells in the CS group compared to that in the CT group (p=0.0044; Kruskal-Wallis and Dunn’s multiple comparisons tests; n=5 for each experimental group). **(B)** There was a significant increase in the number of IL-10^+^ cells in the CS group and the CS/Tol group compared to that in the CT group (p<0.0001, both; one-way ANOVA and Holm-Sidak’s multiple comparisons test; n=5 for each experimental group). The violin plots show the frequency distributions of the analyzed data. **(C)** Photomicrographs of IL-17 immunostaining in the lung parenchyma in the CT, CS and CS/Tol groups (1000x magnification; red arrows show positive cells in brown). **(D)** Photomicrographs of IL-17 immunostaining in the lung parenchyma in the CT, CS and CS/Tol groups (1000x magnification; red arrows show positive cells in brown).

Cigarette smoke caused an increase in the number of IL-17^+^ cells in both exposed groups, but statistical analysis revealed a significant increase in the number of these cells only in the CS group compared to the CT group (**Figure 4A**; p=0.0044). In contrast, the quantification of IL-10^+^ in the alveolar parenchyma showed a significant increase only in the CS/Tol group compared to the CT group (**Figure 4B**; p=0.0034), although this cell type was increased in both the CS and CS/Tol groups. In **Figures 4C**, **D**, the photomicrographs ilustrate IL-17 and IL-10 immunostaining in the lung parenchyma, respectively.

**Figure 5:**
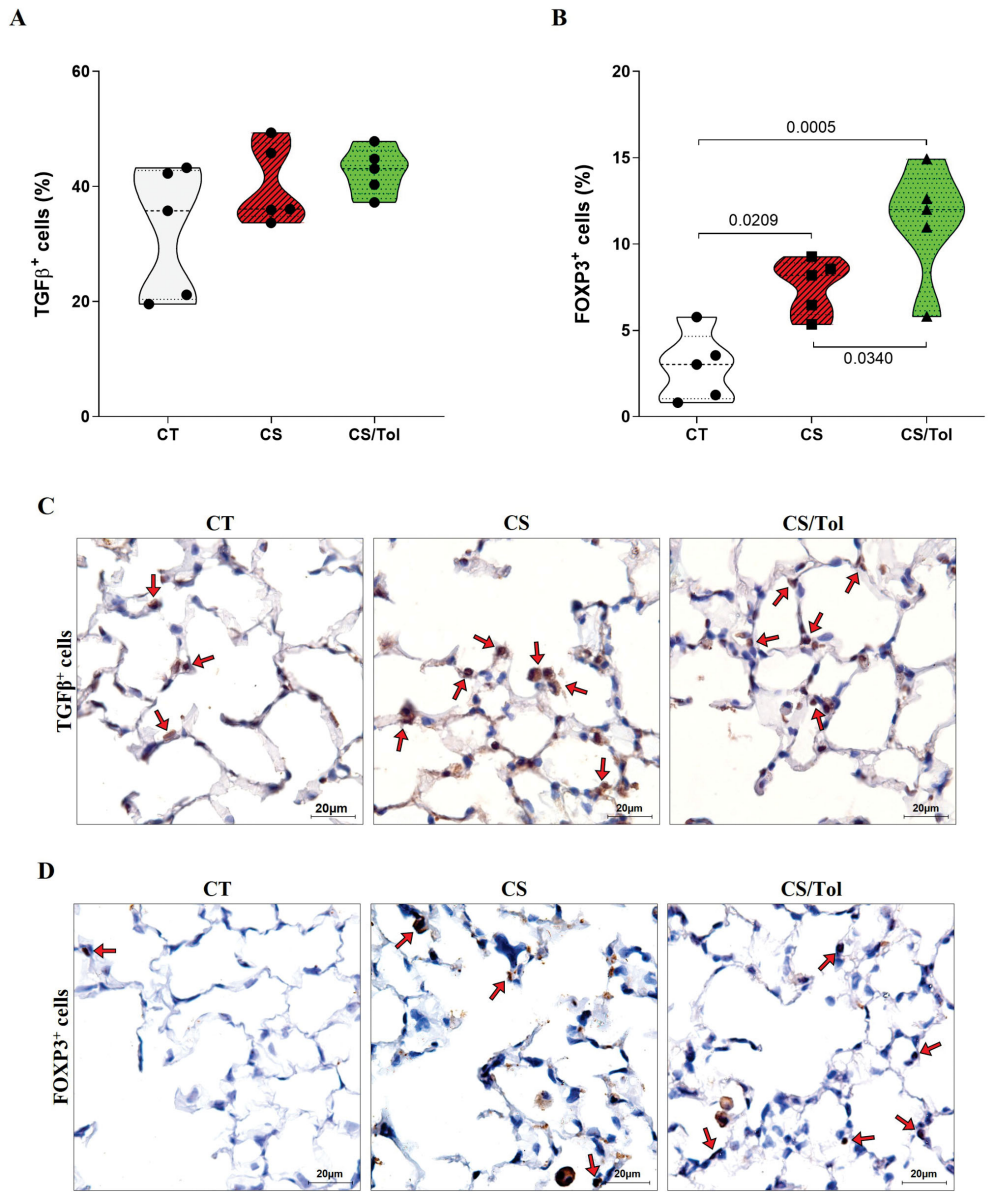
Proportion of TGFβ^+^ and FOXP3^+^ cells in the lung parenchyma and representative photomicrographs of immunostaining for TGFβ^+^ and FOXP3^+^ cells. **(A)** There was no difference in the number of TGFβ^+^ cells between the experimental groups (one-way ANOVA and Holm-Sidak multiple comparisons test, n=5 for each experimental group). **(B)** There was a significant increase in the number of FOXP3^+^ cells in the CS and CS/Tol groups compared to that in the CT group (p=0.0209 and p=0,0005, respectively), and a significant increase in the number of FOXP3^+^ cells in the CS/Tol group compared to that in the CS group (p=0,034, one-way ANOVA and Holm-Sidak’s multiple comparisons test, n=5 for each experimental group). The violin plots show the frequency distributions of the analyzed data. **(C)** Photomicrographs of TGFβ immunostaining in the lung parenchyma in the CT, CS and CS/Tol groups (1000x magnification; red arrows show positive cells in brown). **(D)** Photomicrographs of Foxp3 immunostaining in the lung parenchyma in the CT, CS and CS/Tol groups (1000x magnification; red arrows show positive cells in brown).

Immunostaining of TGFβ^+^ cells did not show a significant difference between the groups for this marker, although there was a slight increase in the groups exposed to cigarette smoke CS and CS/Tol compared to the CT group (**Figure 5A**). Statistical analysis revealed a significant increase in FOXP3^+^ cells in the groups exposed to cigarette smoke CS or CS/Tol compared to those in the CS group (**Figure 5B**; p=0.0209 and p=0.0005, respectively). There was also a significant increase in these cells in the CS/Tol group compared to those in the CS group (**Figure 5B**; p=0.034). In **Figures 5C**, **D**, the photomicrographs ilustrate TGFβ and Foxp3 immunostaining in the lung parenchyma, respectively.

### 3.3 T lymphocyte autoimmunity response to cigarette smoke and Treg differentiation upon Col V-induced tolerance

#### 3.3.1 Cigarette smoke-induced T lymphocyte activation

**Figure 6:**
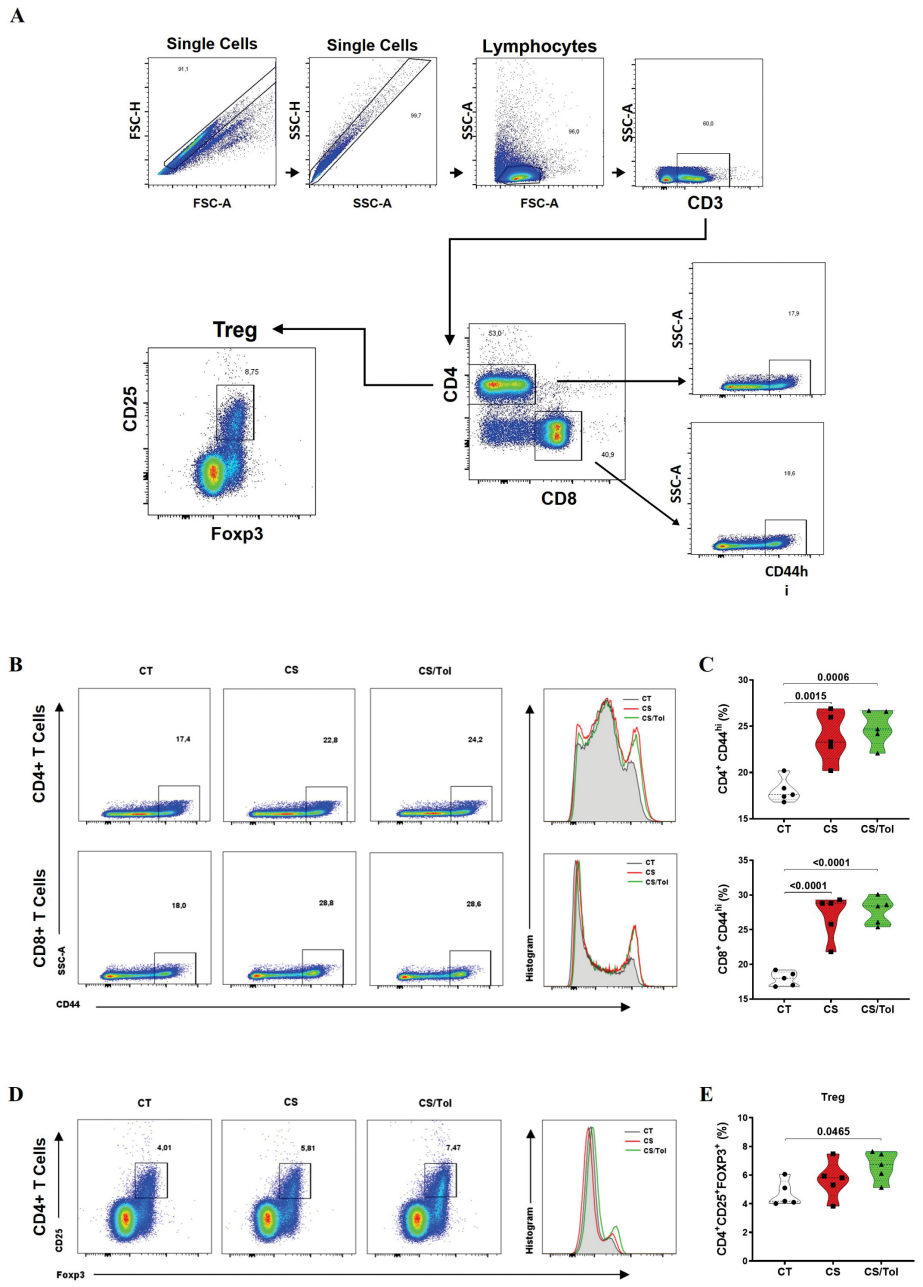
Frequencies of T lymphocyte phenotypes in the spleen (CD4^+^CD44^hi^, CD8^+^CD44^hi^, and CD4^+^CD25^+^FOXP3^+^). **(A)** Gating strategy used for flow cytometry: single cells, CD3^+^ cells, CD4^+^ cells or CD8^+^ cells, CD44^hi^ cells, and CD4^+^ cells, CD25^+^ cells and FOXP3^+^ cells. **(B)** Percentage of CD44^hi^ cells among CD4^+^ and CD8^+^ T cells. **(C)** There was an increase in the frequency of CD4^+^CD4^hi^ cells in the CS and CS/Tol groups (p=0.0015 and p=0.006, respectively) compared to that in the CT group, and there was an increase in the frequency of CD8^+^CD4^hi^ cells in the CS and CS/Tol groups (p< 0.0001, both). **(D)** Percentage of CD25^+^FOXP3^+^ CD4^+^ T cells markers. **(E)** There was an increase in the Treg (CD4^+^CD25^+^FOXP3^+^) frequency in the CS/Tol group (p=0,0465) compared to that in the CT group. (One-way ANOVA and Holm-Sidak multiple comparisons test, n=5 for each experimental group). The violin plots in the graphs show the frequency distributions of the analyzed data.

Analysis of the frequency of systemic CD4^+^ and CD8^+^ T lymphocytes, which are spleen lymphocytes, was performed according to the gating strategy depicted in **Figure 6A**. We first assessed CD44^hi^ expression in CD4^+^ and CD8^+^ T cells. CD44^hi^ expression in the CD4^+^ T lymphocytes of the groups exposed to cigarette smoke CS or CS/Tol was significantly greater than the CT group (**Figures 6B, C**; p=0.0015 and p=0.006, respectively). Analysis of the CD44hi T-cell subpopulations revealed greater CD44^hi^ expression in the CS and CS/Tol groups than in the CT group (**Figures 6B, C**; both p<0.0001).

#### 3.3.2 Tolerance-induced FOXP3^+^ Treg cells

Analysis of the Treg (CD4^+^CD25^+^FOXP3^+^) lymphocyte frequency in the spleen was performed according to the gating strategy depicted in **Figure 6A**. There was a significant increase in the frequency of Tregs in the spleen in the CS/Tol group compared to that in the CT group (**Figures 6D, E**; p=0.0465).

### 3.4 Col V-induced tolerance contributes to a regulatory microenvironment in lung tissue

#### 3.4.1 Cytokine suppression in the lungs of tolerant model mice

The levels of inflammatory cytokines (IFNγ, IL-6, TNF, IL-10, and IL-17A) in the spleen and lung homogenates were determined by flow cytometry (CBA) and corrected for protein dosage. CBA analysis of the cytokine profile in lung homogenates revealed a greater inflammatory profile in the CS group than in the CT group and a significantly lower inflammatory profile in the CS/Tol group than in the CS group.

**Figure 7:**
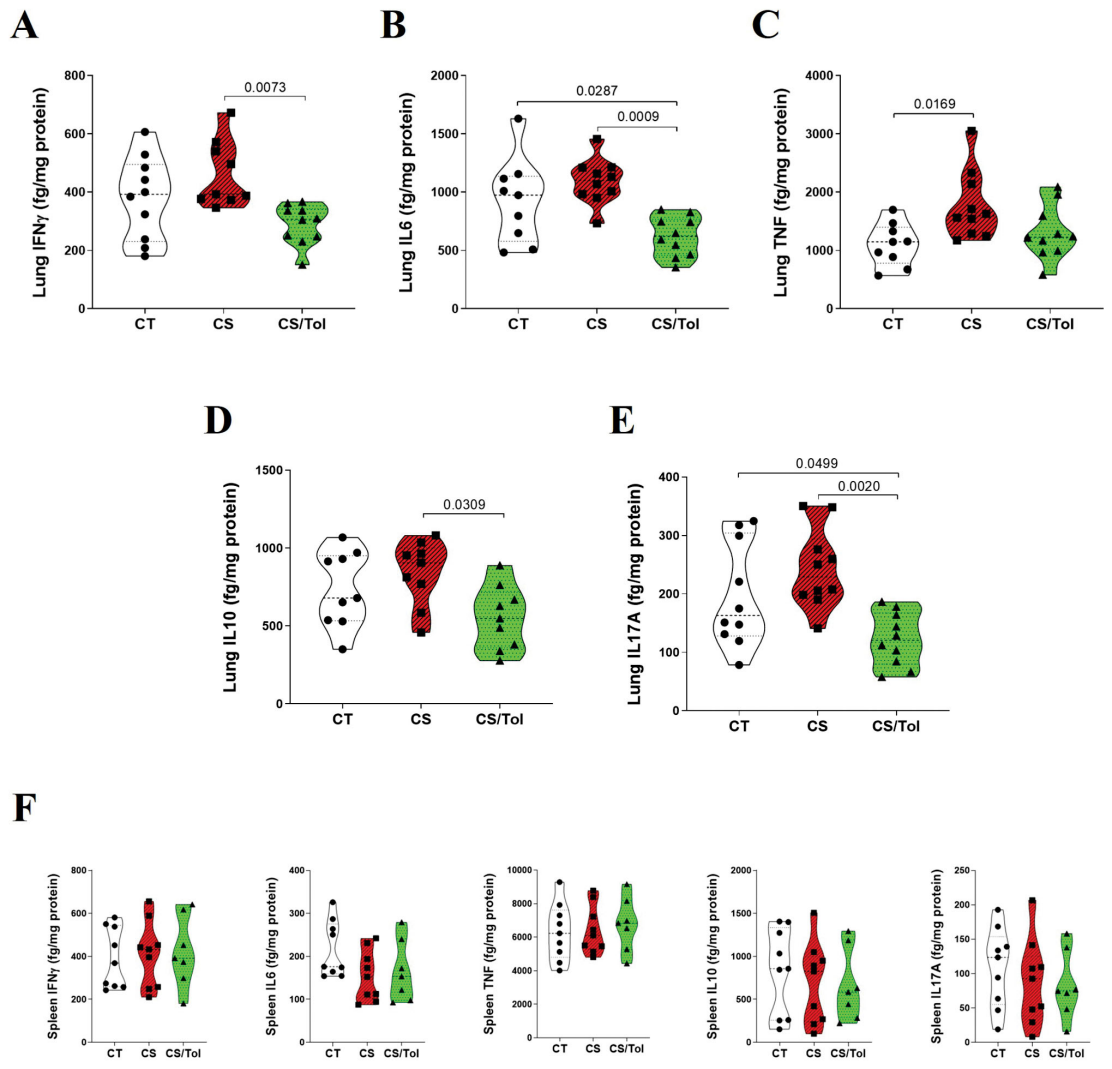
Levels of cytokines in the lung and spleen. **(A)** There was a significant decrease in lung IFNγ in the CS/Tol group compared to the CS group (p=0.0073). **(B)** There was a significant decrease in lung IL-6 in the CS/Tol group compared to the CS group (p=0.0009) and the CT group (p=0.0287). **(C)** There was a significant increase in lung TNF-α in the CS group compared to the CT group (p=0.0169). **(D)** There was a significant decrease in lung IL-10 in the CS/Tol group compared to that in the CS group (p=0.0309). **(E)** There was a significant decrease in lung IL-17A in the CS/Tol group compared to that in the CS group (p=0.002) and CT group (p=0.0499) (one-way ANOVA and Holm-Sidak’s multiple comparisons test, n=9-10 for all experimental groups). **(F)** There were no differences between groups for any of the inflammatory cytokines (one-way ANOVA and Holm-Sidak’s multiple comparisons test, n=9 for CT and CS, n=7 for the CS/Tol group). The violin plots show the frequency distributions of the analyzed data.

In the lung, there was a significant reduction in IFNγ (p=0.0073) and IL-6 (p=0.0009) in the CS/Tol group compared to those in the CS group (**Figures 7A, B**). TNF-α levels were significantly greater in the CS group than in the CT group (**Figure 7C**; p=0.0169). Additionally, there was a significant reduction in IL-10 (**Figure 7D**; p=0.0309) and IL-17A (**Figure 7E**; p=0.002) in the CS/Tol group compared to those in the CS group. The levels of the cytokines IL-6 and IL-17A in the lung were greatly reduced in the CS/Tol group and were significantly lower than those in the CT group (**Figures 7B, E**; p=0.0287 and p=0.0499, respectively). CBA analysis of spleen homogenates revealed no differences between the experimental groups for any of the measured inflammatory cytokines, as graphically demonstrated in **Figure 7F**.

#### 3.4.2 M2-polarized macrophage induced by immune tolerance

**Figure 8:**
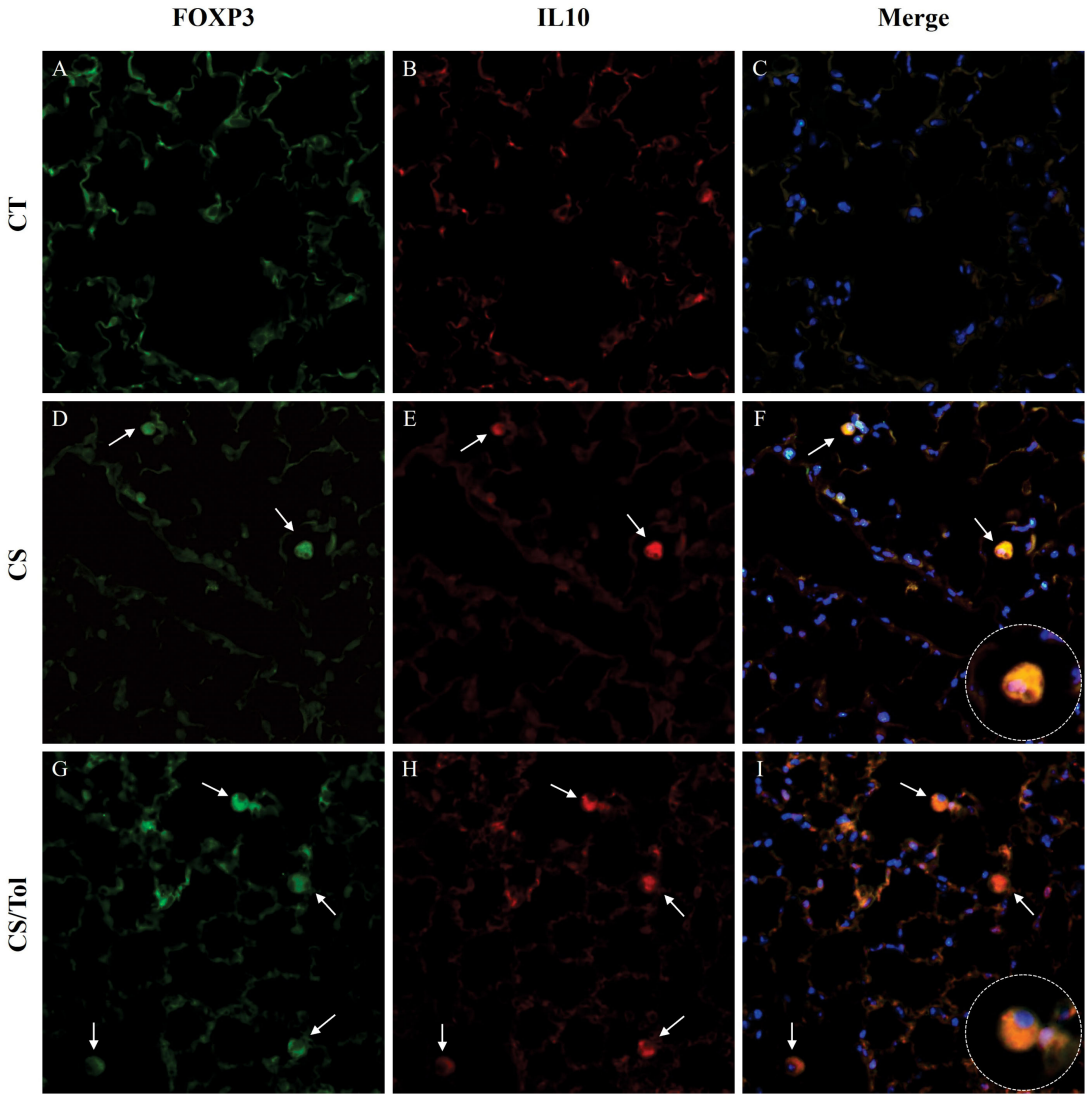
Immunofluorescence images of the lung parenchyma in all experimental groups showing FOXP3 (green) and IL-10 (red) immunostaining and merged images showing colocalization of both markers. The expression of FOXP3 (green) and IL-10 (red) in cells that infiltrated the lung parenchyma of the control, cigarette smoke, and cigarette smoke tolerance groups was assessed by double-label immunofluorescence. Nuclei were stained with DAPI (blue), and white arrows indicate positive cells. 400× magnification.

Immunofluorescence double staining for Foxp3 and IL-10 showed an increase in macrophages positive for these markers in animals from both the CS and CS/Tol groups. However, the tolerant animal from the CS/Tol group had a considerably higher predominance of IL-10 macrophages than the animal from the CS group, implying that immunological tolerance enhanced the polarization towards the M2 profile (**Figure 8**).

## 4 Discussion

We hypothesized that if autoimmunity to Col V contributes to COPD development, then inducing tolerance could mitigate lung damage and remodeling caused by exposure to cigarette smoke. Indeed, we prevented lung ECM changes in a short-term COPD experimental model by inducing nasal tolerance to Col V. We found that mice that received tolerance treatment exhibited notably less structural damage to the alveolar parenchyma and peribronchovascular axis following a month of cigarette smoke exposure. Col V-induced tolerance led to Treg cell accumulation in the alveolar parenchyma. This resulted in suppressive microenvironments in the lungs that hindered the proinflammatory activity of innate and adaptive immune cells in this tissue, preventing emphysema in the tolerant mice.

Regarding structural changes, tolerant mice in the CS/Tol group had Lm values comparable to those of control animals and significantly lower than those of untreated animals in the CS group. Early structural changes induced by cigarette smoke in mice have been previously demonstrated, (43, 44) including an increase in Lm in a 4-week COPD cigarette smoke induction model. (45) The increase in Lm observed in the CS group was accompanied by peribronchovascular edema. Studies conducted in animal models have shown that brief exposure to cigarette smoke can predispose the lungs to inflammation and edema. (46–49) According to previous studies, exposure to cigarette smoke leads to pulmonary endothelial barrier dysfunction and increased permeability in the epithelial layer of the alveolar-capillary membrane, which are likely the causes of the peribronchovascular edema observed. (50, 51) Edema was significantly lower in animals from the CS/Tol group than in those from the CS group, revealing a reduction in this sign of tissue inflammation promoted by Col V-induced tolerance.

An investigation of the lung ECM composition revealed that Col V-induced tolerance mitigated the damage caused by cigarette smoke to collagen fibers, leading to a tendency for an increase in the proportion of Col I in tolerant animals compared to that in untreated animals. However, tolerance treatment did not prevent damage to elastic fibers, although the increase in elastic fiber staining in the alveolar parenchyma was significant only in the untreated group compared to the control group. A reduction in collagen fibers occurs in the large and small airways of patients with mild and moderate COPD and the small airways of unobstructed smokers, (52) and appears to be caused directly by cigarette smoke. (53) Additionally, many studies have shown that elastolysis significantly contributes to the onset of pulmonary emphysema, (54, 55) and an increase in the fraction area of elastic fibers is found in all lung compartments of smokers. (52) Unlike what we observed in the CS group, the pulmonary ECM composition in the CS/Tol group did not change significantly, and the alveolar parenchyma structure remained mostly preserved. Tolerance appears to have had a regulatory influence on ECM turnover (56).

Despite the structural changes observed in the pulmonary ECM of the CS group, we did not observe differences in the respiratory mechanics of these animals, as observed in a previous study. (44) The CS/Tol group also did not show any changes in respiratory mechanics. This is not surprising since functional parameters of respiratory mechanics do not always reflect lung histological changes in animal models, and morphometric parameters are often considered more reliable in detecting the presence of emphysema (57).

An increase in the total number of alveolar inflammatory cells, mostly macrophages, was detected in the BALF from the airways of all animals exposed to cigarette smoke. Tolerance treatment had no impact in this regard, perhaps due to the emphysema induction model we applied, which is marked by repeated aggression caused by cigarette smoke. In experimental models of emphysema, inflammatory infiltration is a frequent finding. (35, 58) Macrophages are the main cell group present in the BALF of mice exposed to cigarette smoke and are the first cell population to increase in number one day after the beginning of exposure. (59) Additionally, in humans, the increase in macrophages in the sputum and lungs of patients with COPD is widely recognized (60).

Moreover, despite not preventing the influx of inflammatory cells, Col V-induced tolerance influenced the progression of the inflammatory process caused by cigarette smoke in the alveolar parenchyma. Immunohistochemical evaluation revealed a trend toward reduced lung macrophage activation (galectin-3^+^ macrophages) in tolerant animals compared to in untreated animals. Galectin-3 is highly expressed in immune cells of all human tissues, (61) is considerably increased in differentiated macrophages (62) and is used mainly as a marker of macrophage activation. (63) Furthermore, intracellular galectin-3 may contribute to the persistence of inflammation by acting as an antiapoptotic factor and promoting the survival of inflammatory cells.Clique ou toque aqui para inserir o texto (64).

Along with interfering with macrophage activation, Col V-induced tolerance suppressed the release of proinflammatory cytokines in the lungs compared to that in untreated mice. In this sense, the tolerant mice did not show the significant increase in TNF-α secretion observed in the CS group. The role of TNF-α in the pathophysiology of COPD is well known. (65) In response to cigarette smoke and other irritants, macrophages release several inflammatory mediators, including TNF-α. (66) Mice without TNF-α receptors do not develop inflammatory infiltrates or ECM breakdown after acute exposure to cigarette smoke, which further reinforces the importance of this factor in the development of emphysema. (67) The observed reductions in macrophage activation and TNF-α release likely contributed to tolerance-mediated preservation of the lung parenchyma.

Macrophages are recognized for their importance in keeping airways free of inhaled particles/pathogens and acting as initiators of the innate immune response. (60) Depending on the stimuli present in the lung microenvironment, macrophages can be polarized into M1 or M2 phenotypes to perform different functions. (68) Exposure to IFN-γ induces the proliferation of M1 macrophages, with roles equivalent to those of T helper (Th1) cells. (69) However, Th2 cytokines such as IL4 promote polarization toward the M2 phenotype. (70) Several prior investigations, both in animal models and humans, have indicated that cigarette smoke exposure causes the differentiation of both M1 and M2 macrophages. (71, 72) Our qualitative evaluation demonstrated that macrophages in both groups of mice exposed to cigarette smoke expressed IL-10 and IL-17. However, the prevalence of IL-10 macrophages was greater in tolerant animals, which suggests that polarization toward the M2 profile is promoted by Col V-induced tolerance, possibly due to the immunosuppressive milieu resulting from immunological tolerance. To corroborate this theory, further investigations, including quantitative assessments of M1 and M2 macrophages in this experimental model, are needed.

Furthermore, Col V-induced tolerance did not affect IL-17 expression in the lung parenchyma; however, the Th17 response in the CS/Tol group was significantly reduced, as evidenced by a marked decrease in IL-17 release in the lung tissue of tolerant animals. Most evidence points to IL-17 as fundamental for mediating inflammation and tissue immunity, acting at the interface between the innate and adaptive systems. (73) Indeed, the activation of innate cellular sources of IL-17A is essential for regulating macrophage accumulation during lung inflammation in mice exposed to cigarette smoke. (74) In contrast, anti-IL-17 antibodies have been shown to reduce inflammation and airway remodeling in a COPD animal model. (75) Furthermore, in our study, tolerance significantly suppressed the release of other proinflammatory cytokines, including IL-6 and IFNγ. In prior work, our group reported that Col V-induced tolerance effectively decreased the inflammatory cellular response in a murine model of bronchiolitis obliterans. (56) Other researchers have found a decrease in systemic levels of proinflammatory cytokines following Col V-induced tolerance via mucosal routes, (27, 33) corroborating our findings.

Along with the suppression of inflammatory responses discussed previously, we observed that regulatory responses were favored both in the lung tissue and systemically in tolerant mice. Col V-induced tolerance promoted a significant increase in the number of regulatory FOXP3^+^ cells and an increase in the number of IL10^+^ cells in the lung parenchyma. To a lesser extent, FOXP3^+^ and IL10^+^ cells were also elevated following subacute exposure to cigarette smoke. Previous studies have demonstrated an increase in Treg cells (CD4^+^CD25^+^Foxp3^+^) in the lungs of mice after four weeks of CS exposure. (76, 77) Additionally, subacute exposure to cigarette smoke can promote an increase in serum IL-10 in mice. (76) These findings suggest that in the early stages of COPD, the immune system attempts to maintain tissue homeostasis by differentiating Treg cells and producing IL-10, thus balancing the inflammatory response. Our findings indicate that tolerance treatment played a substantial role in enhancing this mechanism since in the lung parenchyma, the increase in the IL-10^+^ cell profile was significant only in the tolerant group, and the FOXP-3^+^ cell profile was significantly greater in tolerant animals than in untreated animals. The prevalence of regulatory cells in tolerant animals appears to be critical for protecting the lung parenchyma from injury resulting from cigarette smoke exposure. Moreover, the induction of Treg cell differentiation promoted by Col V-induced tolerance was evidenced by a significant increase in Treg lymphocytes (CD4^+^CD25^+^FOXP3^+^) in the spleens of tolerant animals. FOXP3^+^ regulatory T cells (Tregs) are essential for preserving immunological homeostasis and maintaining self-tolerance (78).

It has been demonstrated that local microenvironments play an essential role in maintaining immune tolerance through tolerogenic cytokines, such as IL-10. (79) However, contrary to our assumptions, the dose of IL-10 in the lung homogenate did not increase the secretion of this cytokine in the lungs of tolerant animals. This finding suggested that immunoregulation was restricted to the microenvironment of the lung parenchyma. Furthermore, peripheral tolerance-induced Tregs may affect the suppression of effector T cells through cell-to-cell contact, (80) not only through cytokine-dependent pathways. In addition to the local effects, immunological tolerance results in a systemic response with peripheral Treg cell induction.

In our experimental model, we considered that the pulmonary inflammation and extracellular degradation triggered by cigarette smoke could expose the immunogenic Col V. By the other hand, the Col V-induced tolerance could protect against ECM lung damage. In this context, would be interesting to considered other extracellular proteins non-collagen as control of the collagen V-intranasal instillation, such as elastin, one of the main proteins in the lung parenchyma degraded as a result of chronic exposure to cigarette smoke. Furthermore, as induced collagen V tolerance-control we could consider the ovalbumin, which induce a murine model of allergic airway disease (81). In addition, we could consider to use as induced tolerance-control the well-known autoimmunity-related protein such as myelin oligodendrocyte glycoprotein involved in autoimmunity in the experimental allergic encephalomyelitis (82). We consider these points a limitation of our study to be considered to future evaluations.

In summary, we found that autoimmunity against Col V may contribute to COPD pathophysiology, as inducing tolerance prevents emphysema progression. As evidence suggests, Col V-induced tolerance promoted Treg cell differentiation systemically and appeared to promote an immunosuppressive microenvironment in lung tissue. The stimulation of Treg cell clonal expansion overcame the effects of Th1 and Th17 cells. Concomitant with these events, the immunosuppressive environment seems to favor M2 macrophage polarization. This contributed to suppressing inflammation in lung tissue, thus preventing injury to the pulmonary ECM caused by exposure to cigarette smoke and, as a result, preventing emphysema development. Our findings indicate that macrophages are critical for this process, but further research is needed to confirm whether Col V-induced tolerance affects macrophage polarization. Although this study has limitations, such as the short duration of cigarette smoke exposure, the results are promising, and further research is needed to confirm our findings and explore the mechanisms underlying the effects of Col V tolerance on COPD lung remodeling. Additionally, further research is needed to explore the applicability of immune tolerance to Col V as a therapeutic approach for COPD.

## Data Availability

The raw data supporting the conclusions of this article will be made available by the authors, without undue reservation.
